# A Modified External Fixation Device for Treating Open Fractures of the Lower Extremities: Two Case Reports

**DOI:** 10.1155/cro/1185255

**Published:** 2026-04-17

**Authors:** Shen Liu, Xiangdang Liang, Songyang Liu, Fei Xie, Zhanshe Guo, Xing Wei

**Affiliations:** ^1^ Department of Orthopaedics, Aerospace Center Hospital, Beijing, China, asch.net.cn; ^2^ Department of Orthopedics, General Hospital of People′s Liberation Army, Beijing, China; ^3^ School of Instrumentation Science and Opto-Electronics Engineering, Beihang University, Beijing, China, buaa.edu.cn

## Abstract

Open fractures of the lower extremities are often poor candidates for early internal fixation due to severe soft tissue injury and high risk of infection. Although traditional external fixation can effectively stabilize fractures and protect soft tissues, it presents several drawbacks, including a bulky structure, poor mechanical stability, inconvenience in dressing and ambulation, and difficulty in combined application with vacuum sealing drainage (VSD). To overcome these limitations, we designed a compact, lightweight modified external fixation device. Its biomechanical stability was enhanced by increasing plate thickness and thread depth. We report two male patients, aged 44 and 56, with open tibial and femoral fractures who underwent debridement and suture followed by stabilization with this modified external fixation device. Following surgery, both patients were capable of early partial weight‐bearing walks without any complications such as pin tract infections, fixation loosening, or nonunion. Radiographic follow‐up demonstrated satisfactory fracture healing, with bony union achieved at 12 and 20 weeks, respectively. The device features a compact structure, facilitating combined use with VSD, and offers ease of operation and high patient comfort. This preliminary experience suggests that the modified external fixation device is a feasible option for selected open lower extremity fractures, though further validation is required.

## 1. Introduction

Open fractures of the lower extremities (tibia and femur) resulting from high‐energy trauma are often unsuitable for primary internal fixation due to severe soft tissue damage, a high infection risk, neurovascular compromise, and the potential for nonunion or delayed healing. External fixation is widely employed as either a temporary or definitive treatment, as it stabilizes the fracture while preserving soft tissue integrity [1],[2]. The external fixation procedure is also relatively straightforward to perform and master compared with internal fixation [3]. Moreover, external fixation causes minimal disruption to the periosteum and local blood supply. Nevertheless, it is often bulky and mechanically less stable, which can lead to unsatisfactory outcomes [4–6], including difficulties with dressing, delayed union or nonunion, and fracture displacement.

Given these limitations, researchers have explored alternatives such as externally applied internal fixation, including the use of locking compression plates (LCPs) as external fixation devices. Although LCP external fixation improves patient comfort and shortens hospital stays [7, 8], its adoption remains limited due to a reduced mechanical stability resulting from an increased plate‐to‐bone distance [9].

To overcome the limitations of both external and internal fixation, we designed a lightweight, highly stable modified external fixation device. This device represents an adaptation of the standard LCP concept. Constructed from titanium alloy, its primary modifications include increased plate thickness and locking thread depth to enhance the rigidity for ex vivo application. The length and number of holes are selected according to the fracture site. The accompanying screws are all self‐tapping locking screws made of titanium alloy, with diameters matching the locking holes of the plate. The detailed design parameters of this device (Table 1), along with the complete biomechanical testing methodology and data, are reported in our previously published study [10]. This report describes two unique cases of open tibial and femoral fractures treated with this modified external fixation device and evaluates its effectiveness and clinical outcomes.

**Table 1:** Design specifications of the modified external fixation device.

Parameter	Specification
Material	Titanium alloy (Ti‐6Al‐4V)
Plate thickness	9 mm
Plate width	7 mm
Hole spacing	14 mm
Locking thread depth	5.5 mm
Screw diameter	4.5 mm (tibia)/5.0 mm (femur)
Manufacturer/fabricator	Beijing Ruilang Medical Equipment Co. Ltd.
Regulatory status	Investigational device (not commercially available; used under ethics‐approved protocol)

## 2. Case Presentation

This study reports two patients (two males, aged 44 and 56 years) with open lower limb fractures who were subjected to debridement and suture plus a modified external fixation procedure (Table 2). The severity of open fractures was assessed using the AO/OTA classification and Gustilo–Anderson classification [11]. The patients had no relevant past medical history, no previous fractures, and were nonsmokers. All patients underwent emergency surgery following a brief preoperative evaluation.

**Table 2:** Patients′ data.

Case	Sex	Age	Cause	Affected leg	AO/OTA classification^a^	Gustilo–Anderson type	Bone union (weeks)	At last observation (months)
1	M	56	Electric saw injury	R	42A2	II	12	6
2	M	44	Traffic accident	L	32A3	II	20	12

^a^AO/OTA classification: 42 = tibia/fibula shaft, A2 = simple oblique fracture; 32 = femur shaft, A3 = simple transverse fracture.

### 2.1. Surgical Procedure

Under lumbar spinal anesthesia, thorough debridement of the open wound on the injured lower limb was performed. The fracture site was exposed through additional small incisions (3–5 cm), and the fracture gap was meticulously cleansed. Anatomical reduction of the fracture was achieved and fixed with lag screws. Soft tissues were closed in layers where feasible, aiming for tension‐free closure. The frame of the modified external fixation was positioned approximately 1 cm above the skin, over the anteromedial aspect of the tibia or the lateral aspect of the femur, to accommodate postoperative soft tissue swelling without impeding the frame. The frame position was determined by two 4.5‐mm‐diameter Kirschner wires fixed at both ends of the fracture. All screw holes were prepared by drilling through a locking sleeve, and all screws were placed with bicortical fixation using a depth gauge. Four or five bicortical locking screws were inserted at either side of the fracture because the AO recommendation of the LCP fixation is to have a minimum of three screws [12]. As part of the postoperative wound management protocol, a VSD dressing was applied over the wound and external fixation frame and removed 3–5 days postoperatively after exudate had diminished. Intraoperative fluoroscopy was used to verify fracture reduction and screw orientation.

To prevent surgical site infection, antibiotic prophylaxis was administered according to established guidelines. Both patients received intravenous antibiotics (cefazolin 2 g every 8 h) for 24 h postoperatively, consistent with AAOS recommendations for Gustilo–Anderson Type II open fractures [13]. No intraoperative cultures were obtained as there were no clinical signs of established infection. All patients were permitted partial weight‐bearing walking on the second postoperative day. After the drainage tube showed no significant exudate (3–5 days), the VSD was removed, and the wounds and screws were cleaned daily with 75% alcohol. Patients were instructed to have sutures removed approximately 2 weeks after surgery and to return for follow‐up x‐ray examinations of the fracture site every 4 weeks.

### 2.2. Case 1

**Figure 1:**
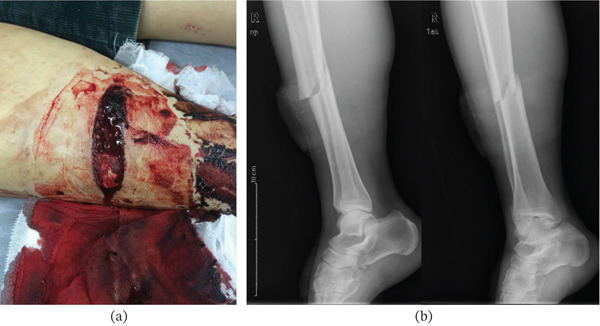
(a) An adult patient sustained an open tibial fracture by a chainsaw splash. (b) Preoperative radiographs: lateral (left) and oblique (right) views showing a simple oblique fracture of the tibial shaft (AO/OTA 42A2). The oblique view was obtained due to patient positioning constraints.

**Figure 2:**
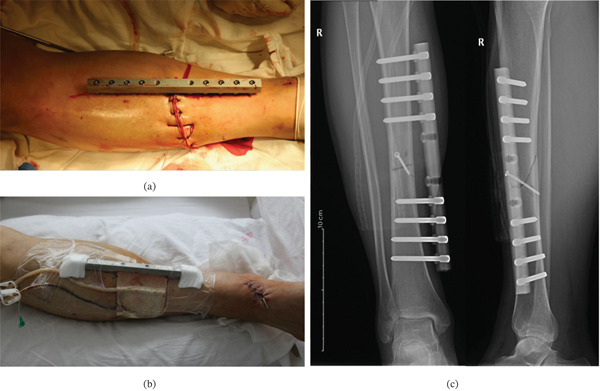
(a) The tibial fracture was stabilized with the modified external fixation and the wound was sutured. (b) The frame and wound were covered by VSD. (c) Anteroposterior radiograph obtained on postoperative Day 1 showing fracture stabilization with the modified external fixation device and lag screws.

**Figure 3:**
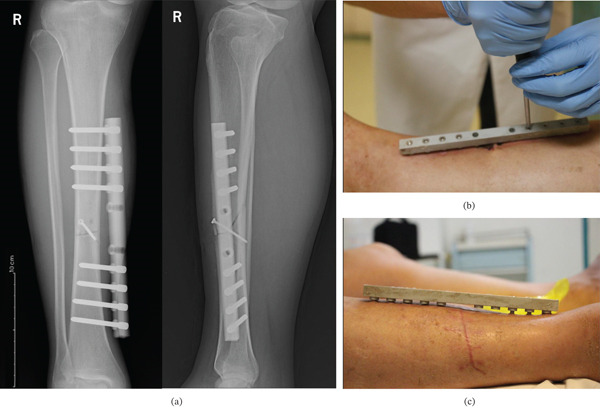
(a) Anteroposterior (left) and lateral (right) radiographs of the tibia at the 4‐week follow‐up showing early callus formation. (b, c) Removal of the two screws closest to the fracture site 1 month postoperatively.

**Figure 4:**
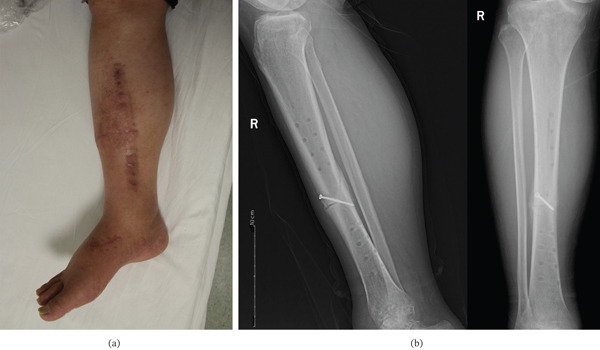
(a) The modified external fixation removed at the 3‐month follow‐up. (b) Posteroanterior radiographs at the 12‐week (3‐month) follow‐up after device removal: lateral view (left) and anteroposterior view (right) demonstrating healed fracture with bridging callus across all four cortices. The modified RUST score was 13, confirming clinical union.

**Figure 5:**
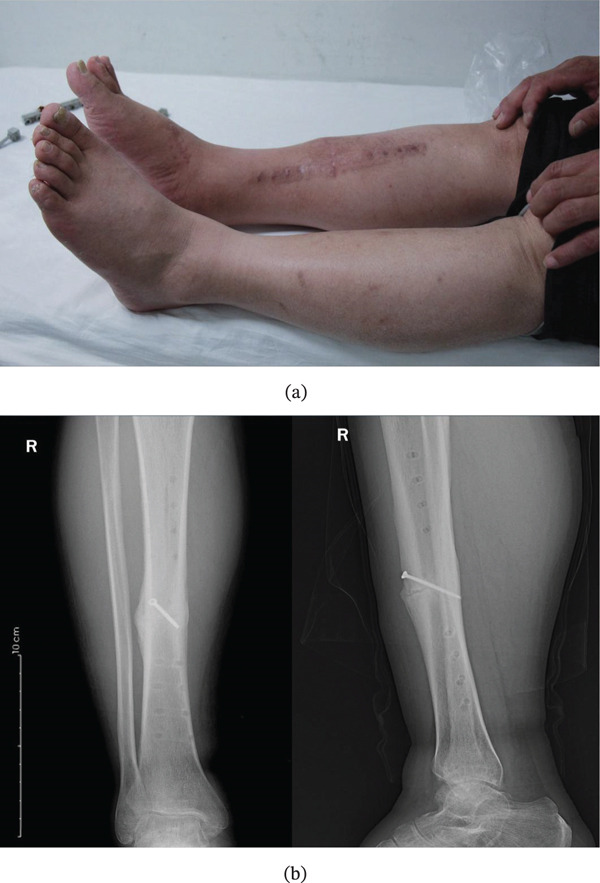
(a) At 6 months postoperatively the American Orthopedic Foot and Ankle Society (AOFAS) score reached 91 points. (b) Anteroposterior (left) and lateral (right) radiographs at the 6‐month follow‐up showing complete bony union with remodeling.

A 56‐year‐old adult patient sustained severe injuries caused by a chainsaw splash, resulting in an open fracture of the right tibia and fibula (42A2, Gustilo–Anderson Type II) (Figure 1). The tibialis anterior muscle was involved, leading to exposure of soft tissue measuring 10 × 2 cm. Upon admission, the patient underwent emergency surgery (debridement and suture + modified external fixation) within 6 h postinjury (Figure 2). No postoperative wound infection was observed. At the 4‐week follow‐up, tibial radiographs revealed callus growth and cortical bone bridging. At this stage, the two screws closest to the fracture site could be removed (Figure 3). Subsequently, screws on both sides of the fracture were gradually removed during follow‐up visits based on the progression of fracture healing. At 12 weeks postoperatively, x‐rays showed a large amount of callus surrounding the fracture ends, with a modified Radiographic Union Score for Tibial (mRUST) fractures score of 13/16, reaching clinical union, and all screws and external supports had been removed (Figure 4). At the 6‐month postoperative outpatient follow‐up, radiographs revealed a further increase in callus formation with complete disappearance of the fracture line. Moreover, the American Orthopedic Foot and Ankle Society (AOFAS) score reached 91 points (Figure 5). The patient was able to walk with full weight‐bearing without pain and expressed satisfaction with the entire treatment process.

### 2.3. Case 2

**Figure 6:**
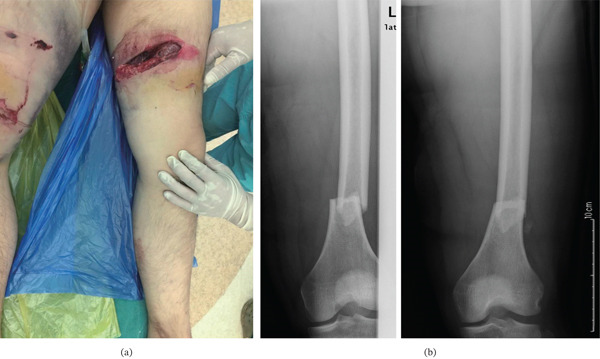
(a) A male patient suffered the open femoral shaft fracture due to a traffic accident. (b) Preoperative radiographs: oblique (left) and anteroposterior (right) views showing a transverse fracture of the left femoral shaft (AO/OTA 32A3). The oblique view was obtained due to patient positioning constraints.

**Figure 7:**
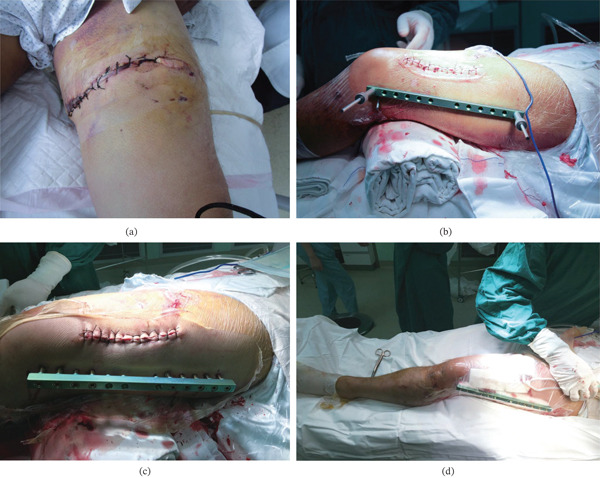
(a) The original wound following debridement and suturing. (b, c) An incision was made on the lateral aspect of the thigh, followed by fracture reduction and fixation with lag screws. After wound closure, a modified external fixation was applied. (d) The external fixator frame and the incision were covered with a VSD system after operation.

**Figure 8:**
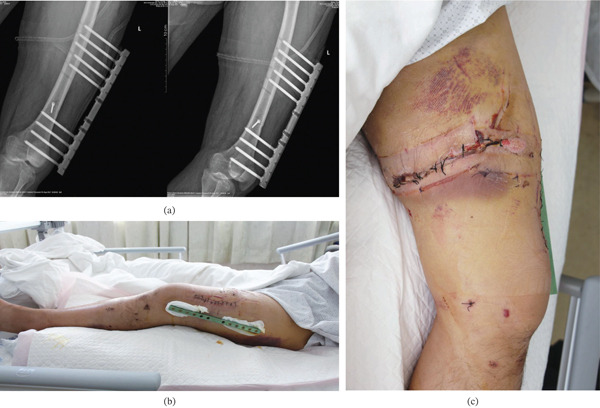
(a) Anteroposterior radiograph on postoperative Day 3 demonstrating anatomical reduction and stable fixation with the modified external fixation device and lag screws. (b, c) The incision and external fixator after VSD removal at 3 days postoperatively; no significant pin tract reaction was observed.

**Figure 9:**
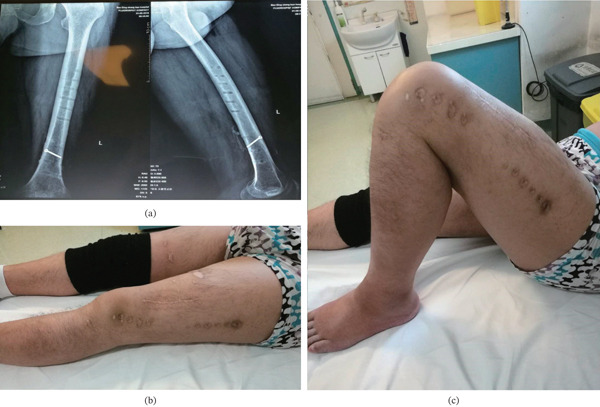
(a) Anteroposterior (left) and lateral (right) radiographs of the left femur obtained at the 20‐week postoperative follow‐up, demonstrating solid bony union with complete disappearance of the fracture line. The modified RUST score was 16, confirming bridging across all four cortices. (b, c) The surgical incision was well‐healed, and the range of motion at the knee was extension 0° to flexion 130°.

A 44‐year‐old male patient suffered a left femoral shaft fracture (32A3) due to a traffic accident. A transverse laceration, approximately 20 cm in length, was observed on the anteromedial aspect of the thigh, without active bleeding but with significant contamination (Gustilo–Anderson Type II) (Figure 6). After evaluation, wound debridement and suture were performed under anesthesia. Subsequently, a modified external fixation procedure was conducted through a surgical incision approximately 5 cm in length, centered over the fracture site on the lateral aspect of the thigh, avoiding the original wound, to reduce the fracture. Postoperatively, VSD and antibiotics were administered for infection prevention (Figure 7). The VSD system was removed on postoperative Day 3 when no significant wound exudate was observed. Routine dressing changes were performed regularly. Postoperative x‐rays demonstrated satisfactory fracture reduction, and the patient was allowed to ambulate with partial weight‐bearing (Figure 8). Of note, the most proximal screws in this construct were placed in a unicortical configuration due to the limited length of the screws. This was a deliberate intraoperative decision based on anatomical constraints. Biomechanical testing from our prior study confirmed that this configuration provides sufficient stability for full weight‐bearing in adults. Moreover, the controlled micromotion permitted by unicortical fixation may have contributed to the robust callus formation. Serial radiographs confirmed no evidence of progressive screw migration or loosening throughout the follow‐up period. At the 20‐week follow‐up, anteroposterior and lateral radiographs demonstrated satisfactory fracture healing with disappearance of the fracture line, reflected by a mRUST score of 16/16 (indicating a solid union across all four cortices), while the position of the lag screws remained unchanged (Figure 9A). The range of motion at the knee was extension 0° to flexion 130° (Figure 9B,C). At 1‐year postoperative follow‐up, the patient had an excellent functional result and was fully weight bearing with a well‐healed femur. The Lower Extremity Functional Scale (LEFS) score was 72/80, indicating minimal functional limitation.

No adverse events (e.g., pin tract infection, implant loosening, nonunion, neurovascular injury, or thromboembolic events) were observed in either patient during the follow‐up period. No unanticipated events related to the device or surgical procedure occurred. Both patients tolerated the treatment well and completed the planned follow‐up schedule without deviation (except for Case 1 being lost to follow‐up after 6 months).

**Figure 10:**
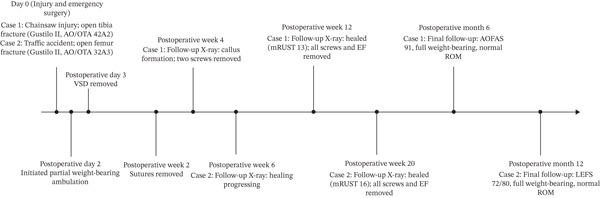
Combined timeline of clinical course for both patients. Key events from injury to final follow‐up are presented for Case 1 (tibia fracture) and Case 2 (femur fracture).

A combined timeline of the clinical course for both patients is presented in Figure 10.

## 3. Discussion

Due to concerns regarding the risk of postoperative soft tissue infection and osteomyelitis, external fixation has consistently been the preferred method for either initial or definitive treatment of lower extremity open fractures classified as Gustilo–Anderson Type II or above [6]. This technique is straightforward to apply, readily adjustable, and requires only minimal incisions. Furthermore, it avoids additional disruption to the blood supply and does not necessitate soft tissue stripping [14, 15]. However, the drawbacks of traditional external fixation cannot be overlooked. Firstly, traditional external fixation, with its connecting rods and clamps, provides relatively low fixation strength. Long‐term use may lead to unstable fracture fixation, a finding supported by our biomechanical experiments [10]. Secondly, patients often express dissatisfaction due to the bulky and uncomfortable nature of the external fixation device. Thirdly, the relatively large diameter of the fixation pins is associated with a higher risk of postoperative infection, requiring substantial time and effort from both medical staff and patients for pin tract care. Furthermore, the coordination of external fixation with VSD is challenging. Traditional external fixation is difficult to use in conjunction with VSD, as the frame and pins may interfere with the seal, leading to poor adhesion and air leakage [16], thereby increasing the risk of wound infection.

To address these shortcomings, externalized internal fixation, particularly the use of LCP, has emerged as an alternative to traditional external fixation [17]. Marti and Besselaar [18] first introduced AO plates as external fixation in 1984, demonstrating their efficacy for treating forearm and tibial fractures. Ramotowski later described the external application of LCPs for open tibial fractures, noting that their lower profile and removability improved patient acceptance and facilitated postoperative rehabilitation [19]. Similarly, Hidayat et al. [20] reported satisfactory fracture union and wound healing in five patients with Gustilo Grade III tibial fractures managed using E‐LCP. However, when used externally, LCP stability might be greatly reduced. Ahmad′s research shows that the axial compressive strength of LCP was decreased 63% when placed 5 mm away from the bone surface [9]. Consequently, some researchers have applied the femoral Less Invasive Stabilization System (LISS) for distal tibial fractures to enhance fracture stability [21]. However, since the distal femoral LCP is specifically designed for internal fixation, its use as an external fixation device in clinical practice raises certain ethical concerns, which limit its widespread application [22]. Moreover, potential concerns regarding the mechanical strength of the femoral LCP when used externally may exist. To date, no case reports have documented the use of femoral LCP as an external fixation device for femoral fractures.

We have designed a modified external fixation device, which enhances stability by modifying the dimensions of the fixator and the depth of the locking threads based on the LCP. Preliminary finite element analysis and mechanical testing were conducted to evaluate its mechanical properties. The results demonstrated that in axial compression testing, the compressive stiffness of the device reached 519.489 N/mm, which was significantly higher than that of the traditional unilateral external fixation device (327.153 N/mm) and the externalized LCP (316.763 N/mm). Under an axial load simulating the average adult body weight (~700 N), the maximum displacement at the fracture site was 1.206 mm. This value is below the upper threshold of 2 mm that is required for fracture healing, thus facilitating bone union [10]. In the context of femoral fracture application, two additional screws were incorporated to ensure enhanced structural stability.

This modified device demonstrates several unique advantages: (1) By adjusting the size of the fixator and the depth of the threads, the strength of the device is significantly enhanced, thereby substantially improving fracture stability. In this study, patients were able to perform weight‐bearing walking early postoperatively. (2) The device is convenient to operate, and the procedure is similar to internal fixation. After fracture reduction, screw insertion using an electric drill can save operative time and is more convenient compared to external fixation procedures. Meanwhile, unlike internal fixation, it does not require extending the incision, dissecting tissues, or compromising blood supply. (3) During postoperative follow‐up, screws are removed progressively based on fracture healing status, which can gradually increase axial stress at the fracture site and promote fracture healing. In Case 1, x‐ray images at 12 weeks postoperatively showed that the fracture line had essentially disappeared, with increased callus formation and evident bone remodeling. (4) The compact size of the device helps improve patient comfort, allows it to be concealed under clothing, and preserves the mobility of adjacent joints postoperatively.

In our cases, the VSD system was applied directly over the wound and the modified external fixation device. Although there is currently insufficient evidence for the potential benefits of negative pressure wound therapy (NPWT) in the treatment of open fractures [13], we observed significant practical benefits of our low‐profile device: its compact, skin‐contoured design facilitated seamless application of the VSD foam and adhesive drape, effectively minimizing air leakage commonly encountered around conventional external fixation pins. This technical compatibility may enhance the feasibility of using NPWT when clinically indicated, without claiming additional biological benefits.

In our cases, the VSD system was applied directly over the wound and the modified external fixation device, demonstrating good compatibility. VSD, with its ability to provide continuous postoperative negative pressure for the drainage of secretions, is beneficial to maintain wound cleanliness and promote the growth of granulation tissue [23]. The device has a simple structure and is close to the skin, which facilitates foam and adhesive plastic coverage. Furthermore, it can effectively avoid air leakage around the pins and overcome the difficulty of operating in conjunction with external fixation.

It is important to acknowledge the limitations of this study. As a case report series, the small sample size (*n* = 2) restricts the generalizability of the findings and precludes definitive conclusions regarding the efficacy and safety of the modified external fixation device. Another limitation of this report is the lack of longer term (≥ 12 months) follow‐up data for Case 1 due to loss to follow‐up 1 year postoperatively. In clinical practice, loss to follow‐up among trauma patients represents a common challenge for prospective studies. In the two cases of open fracture reported in this study, the concomitant use of lag screws may have influenced the independent evaluation of the modified external fixation device and potentially introduced bias. While this approach aligns with established principles for managing intra‐articular or rotational unstable fracture patterns, it precludes attribution of the healing results solely to the external fixation device. The lag screws address specific fracture morphology (e.g., rotational control), whereas the external device provides overall axial and torsional stability. This distinction is critical when interpreting the device′s independent contribution to fracture healing.

## 4. Conclusion

In summary, this modified external fixation device achieved satisfactory clinical outcomes in the two patients treated. These two cases demonstrate its potential for effectively managing open fractures. Further studies, including rigorous randomized controlled trials, are required to further establish its efficacy. Furthermore, given its minimally invasive nature, portability, ease of removal, ability to promote fracture healing, and low risk of infection‐related complications, this device may also have potential applications in closed lower limb fractures, meriting further investigation.

## Funding

This study was supported by the Science Foundation of AMHT (2022YK23).

## Ethics Statement

This study was approved by the Ethics Committee of Chinese PLA General Hospital (Approval No. S2021‐337‐01), where both patients were treated and the research was conducted. All procedures were performed in accordance with the ethical standards of the 1964 Declaration of Helsinki and its later amendments. This study was not prospectively registered, as it represents a preliminary case series of an investigational device under an ethics‐approved research protocol, rather than a formal clinical trial requiring registration.

## Consent

Written informed consent was obtained from both patients for publication of this case report and any accompanying images. A copy of the written consent is available for review by the editor‐in‐chief of this journal upon request.

## Conflicts of Interest

The authors declare no conflicts of interest.

## Data Availability

The data that support the findings of this study are available from the corresponding authors upon reasonable request.
